# Discussing blockchain applications in TED Talks: A fashion wave approach to understanding the blockchain phenomenon

**DOI:** 10.1371/journal.pone.0289025

**Published:** 2023-07-25

**Authors:** Riccardo Bonazzi, Heidi Gautschi, Gianluigi Viscusi

**Affiliations:** 1 University of Applied Sciences and Arts Western Switzerland (HES-SO), Sierre, Switzerland; 2 Haute École Pédagogique Vaud (HEP-Vaud), Lausanne, Switzerland; 3 Linköping University, Linköping, Sweden; 4 Imperial College Business School, London, United Kingdom; University of Victoria / Universiti Teknologi Malaysia /, CANADA

## Abstract

Since its first appearance as the infrastructure supporting Bitcoin, blockchain has received different waves of attention from practitioners and academics. Besides the degree of interest, the attention to blockchain has often focused not merely on its own potential and characteristics but on its applications field. However, looking at its application and deployment in domains other than cryptocurrency or the nascent non-fungible tokens (NFTs), blockchain technology does not seem to be widely adopted or is still in its early stages. Given this, we are interested in how managers and the general public are informed about new technology other than through academic papers or the specialized press. In this paper, we analyze how blockchain has been presented by speakers invited to give TED and TEDx Talks to identify the most common terms used to present blockchain in these talks using a multi-methods approach (qualitative analysis and big data analysis) and see if the discourse surrounding blockchain has shifted over time. The results of this study show how the different perspectives brought by broadcasters like TED often overshadow a technological innovation like the blockchain in its evolution and application by the fact that the focus is instead shifted to products and services built on it. Also, this study shows how different degrees of attention and expertise are associated with each fashion wave of new or emergent technology innovations.

## Introduction

Since its first appearance as the infrastructure supporting Bitcoin [1–3], blockchain has received different waves of attention from practitioners and academics. Besides the degree of interest, the attention to blockchain has often focused not merely on its own potential and characteristics but on its applications field [4]. These latter range from providing new solutions based on decentralized architectures for incumbent technology and platforms like online social media [5] to effectively contributing to “address problems that afflict our society, making it more inclusive, fair, and resilient” [6, p. 417] such as, in the case of use of blockchain for humanitarian purposes [7] or its application in support of the United Nations (UN) Sustainable Development Goals–SDGs [8]. However, it can be argued that most managers and decision-makers, as well as general users, do not yet fully understand blockchain and its application field [9], although there is a growing interest in investing in the technology [10].

Considering this, we are interested in how o managers and the general public are informed about new technology other than through academia or the specialized press. This subject has been studied by considering the role of technology advisors and Industry analysts, for example, [11–14]. In this paper, we analyze how blockchain has been presented by speakers invited to give TED and TEDx Talks. These talks are understood to be vectors sharing specialized content in an accessible way. Accordingly, the paper aims to identify the most common terms used to present blockchain in these talks and see if the discourse surrounding blockchain has shifted over time.

These talks have been considered the substantive domain for our investigation due to their broad diffusion and the attention that the various events organized by TED rise globally, especially for technology and innovation topics. TED stands for “technology, entertainment, design.” The impetus for the conference was a recognition that these three areas were converging. Technological innovation has been prominent since the first TED conference in 1984. The 1984 lineup included Mickey Schulhof of Sony demoing the compact disc (CD) and Nicholas Negroponte giving five technology predictions.

While the TED organization now offers more than just talks, the talks are what they are best known for. People present official TED talks at a limited number of venues, either at one of the two leading conferences or the TED global conference. This gives these conferences an aura of exclusivity. Not just anyone can attend, and not just anyone can present at these venues [15]. There is a selection process for choosing TED talkers and audience members to maintain quality and diversity [16]. TED Talkers are recognized experts in their field. For example, Don Tapscott, Mark Schwartz, Bettina Warburg, and Neha Narula have all given TED Talks on various aspects of blockchain. Their talks are part of our initial analysis. TEDx events are loosely affiliated with TED, and the best TEDx Talks appear on the official ted.com website. TEDx Talks are also available on a dedicated YouTube channel. While TED conferences are global in scope, TEDx events draw on local expertise. TEDx organizers select local experts based on a selection process reflecting both local and global preoccupations. In keeping with TED’s slogan, “ideas worth sharing”, TEDx events serve to promote ideas to a local community. In summary, the analysis presented in this paper aims to contribute to increasing understanding of the relationship between the communication of a technological innovation, like blockchain, and its adoption. This communication may capture not only the attention of practitioners or the public but also influence its eventual application and deployment.

The paper is structured as follows. First, the background literature and the motivations for the study are discussed before the outline of the research methods. We then present the results of the analysis of the TEDx Talks retrieved from YouTube. Finally, a discussion of the contributions and limitations of the paper precedes the concluding remarks and the summary of future work.

## Background

### Blockchain waves of diffusion and application

Blockchain can be considered one of the key technology trends of the last decade that, similarly to other waves in the information systems [17], has captured the attention of managers, decision-makers, and academics with different degrees of enthusiasm and hype. Additionally, blockchain has been studied in various domains like open science, agri-food, healthcare, supply chain management, and energy, among others [18–23]. However, the constant and increasing diffusion of the terms and associated concepts in academia and industrial projects are not necessarily complemented by a similar level of adoption and the number of applications in practice [24,25]. The exceptions are supply chain, with notable players like Walmart and JD [26], cryptocurrencies [27], banking [28], and recently, non-fungible tokens–NFTs [29–31]. Among the topics related to blockchain characteristics, governance has gained the interest of academics [32–35], especially about the actual application of blockchain as a governance infrastructure in sensitive domains like the public sector, which nonetheless appears far from broadly deployed [36]. The adoption of blockchain has also been studied for its potential benefits and challenges for open science [37], agri-food [38,39], healthcare [40], supply chain management and energy [41], among other fields. However, it can be inferred from the current literature on the application and deployment in domains other than supply chain, cryptocurrency, or the nascent NFTs that blockchain is not widely adopted and, in some cases, is still in its early stages [42]. This, notwithstanding the hype the technology has received in the media and from technology advisors.

Taking the above issues into account, blockchain technology seems worth investigating using the theoretical perspective of management fashion [43], questioning, “When and by what process are technically inefficient innovations diffused or efficient innovations rejected?” [44, p. 587]. A management fashion is “a relatively transitory collective belief, disseminated by management fashion setters, that a management technique leads rational management progress” [43, p. 257] and results from a management fashion setting process “by which management fashion setters continuously redefine both theirs and fashion followers’ collective beliefs about which management techniques lead rational management progress.” [43, p. 257]. The literature on management fashion has pointed out the crucial role of supply-side actors and organizations (for example, consulting firms, publishers, business schools, industry associations, and “gurus”) engaged in broadcasting and promoting management innovations, new practices, and technologies [45]. However, the digital environment, where the broadcasting of a technology such as a blockchain happens, has characteristics that challenge the traditional gatekeeping role of supply-side actors and organizations. Innovators can rely on social media and venues like TED, thus, bypassing traditional media and outlets that characterized lifecycles of management fashions pre-digitalization [45,46].

Among social media, Twitter has been one of the main channels for understanding blockchain technology’s perceived usefulness and ease of use, essential elements, and benefits [47,48]. Considering the broadcasting role of social media, Twitter has been used to investigate the trending domains of adoption for the blockchain [49]. Twitter has also been analyzed to understand the communication strategies used for blockchain’s legitimation, as in the case of the study by Rosati et al. [50]. The focus has been on the effort to build legitimacy about Bitcoin and blockchain by business actors with a vital role in the blockchain ecosystem, like the media, firms in the Information Technology (IT) sector, financial services, and consulting firms. Compared to this and similar other state-of-the-art research focused on social networks, in this paper, we focus our attention on TED. The organization is a different player in broadcasting blockchain as an information systems/management fashion wave, with specific characteristics that enable communication and pitching [51–53] of science and technology innovations.

### Science communication, public speaking, and the public sphere

Science communication plays a role in setting the agenda for the scientific community and the general public by circulating what the community considers to be its most important discoveries and applications. Science communication can also attempt to set the agenda differently by getting the public interested in specific ideas, discoveries, and technologies that have, up until then, either been rejected or not picked up by the scientific community [54]. Then, communication “occurs simultaneously at different levels which continuously exert reciprocal influence on one another” [55, p. 384]. Given the reciprocity of science communication, the Internet creates opportunities for greater interactivity between scientists and different publics [56]. TED talks are an example of a number of issues raised by science communication scholars. One crucial question relates to how science and technology are represented via TED talks and TED talkers. And once a scientific discovery or innovation enters the public arena, the public then plays a role in the scientific arena [55,57,58]. TED’s practices and arrangements serve as conditions for the selection of what people actually consider to be scientific expertise, with a possible consequent impact on how policymakers decide which projects are worth funding [59]. TED acts as a marketer for scientists and technologists by coaching speakers to deliver the most impactful speech possible and by selecting the topics to be “promoted” to a larger public [60], thus making TED a medium for science communication that nonetheless should be questioned considering the above-mentioned management fashion waves and the resulting exhibition of science or technology innovation worth promoting. Based on these issues, in this paper, we specifically consider blockchain technology as a subject of communication through TED talks.

Finally, science communication and the development of management fashion waves happen in media contributing to the public sphere’s makeup, which nowadays has a strong digital component. The concept of the public sphere can be applied to analyzing public speaking acts, such as TED talks. According to Habermas [61], the public sphere is both a physical and metaphorical space where public opinion is formed outside of the structure of the State, and the private sphere is represented by the family. It is an intermediary space. The public sphere can be conceived of as a network of people, physical places, and media outlets that circulate ideas that are debated in a rational and critical manner. In short, the public sphere is “the space for reasoned communicative exchange” [62]. The TED organization and its affiliated programs can be considered a symbolic/mediated public sphere [63] since most of the interaction between members of this public occurs online. In fact, the TED website is set up to encourage this type of internet-mediated participation.

## Method

**Fig 1:**
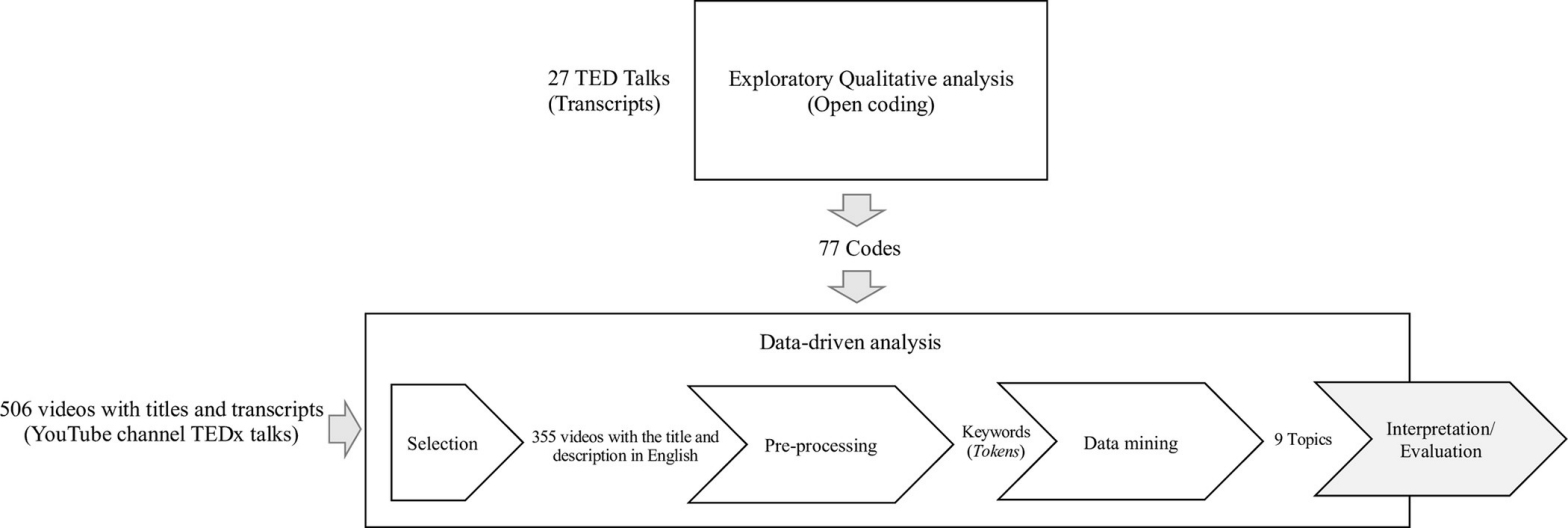
Steps of the research method followed for this study with primary input data sources and outputs.

The study adopts a multi-methods approach in the Internet-mediated research [64,65], whose different steps are shown in Fig 1 and discussed in what follows. In the context of this paper, multi-methods implies that “approaches or methods are used in parallel or sequence but are not integrated until inferences are being made” [Pat Bazeley cited by the authors of 66 on page 119]. Accordingly, in this paper, data are not integrated but are compared in descriptive findings; thus, the study adopts a multi-method and not a mixed-methods approach, where integration is an essential characteristic of [67].

Taking the above issues into account, first, two of the authors conducted an exploratory interpretive qualitative analysis [68] of the selection of talks available on the official TED website using the search query “blockchain.” The final sample retrieved from the website was 27 talks held from 2013 to 2022. For each talk, the transcripts were downloaded, analyzed, and open-coded [69,70] by two of the authors individually and through weekly meetings of 1 hour each in April and May 2022. The two authors involved in this step used Atlas.ti and NVIVO, respectively, which resulted in 77 codes. These codes were added during the joint sessions to an Excel file together with the associated TED Talk data. Being related to an analysis of the flagship TED Talk, the open codes form the basis for the further discussion of the data-driven analysis [71] carried out by the other author on all the TEDx Talks available on YouTube. The choice to move to a data-driven analysis [72] was related to the more significant number of cases (TEDx Talks) to analyze (as mentioned in what follows, 355 videos).

TEDx events are loosely affiliated with the TED organization. While TED does not have direct oversight on the content of independently organized TEDx events, there are strict guidelines regulating these events. TEDx events broadcast a minimum of one official TED Talk, and organizers must attend an official TED conference. TEDx Talks are echo chambers for TED. The details of the data-driven analysis carried out on the TEDx Talks, and its results are discussed in Section 4. Here it is worth noting that the data-driven analysis results were discussed in weekly meetings of 1 hour each during June 2022 with the other co-authors, also considering the open codes identified during the qualitative analysis of the official TED Talks. In summary, we anticipate here that the TEDx themes identified through the data-driven analysis will resonate with talks that are on the official TED website. Furthermore, the data-driven analysis provides results not only similar to what are identified as relevant codes in the qualitative analysis of the official TED Talks but, as we will see in the following sections, also allowed us to identify the relevance of the topics made up of those codes.

## Results

To perform our analysis, we followed the steps suggested by the knowledge discovery in databases (KDD) process: selection, pre-processing, transformation, data mining, and interpretation/evaluation [73].

### Data analysis and pre-processing

**Fig 2:**
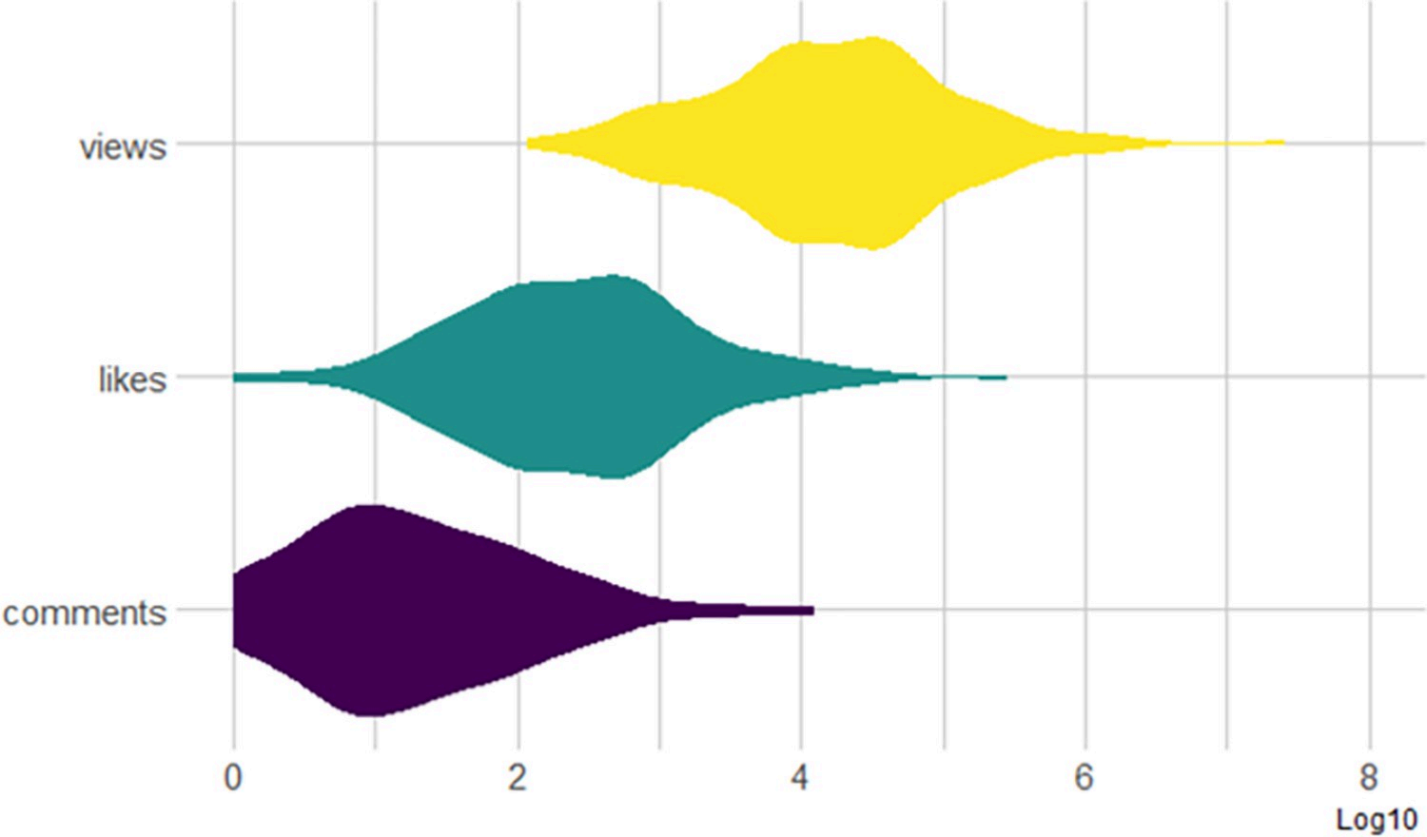
Distribution of views, “likes,” and comments among the 355 YouTube videos.

We obtained the input data in this study by using the YouTube API v3 (for more information, see https://developers.google.com/youtube/v3), searching for “Blockchain” videos on the YouTube channel TEDx Talks (the permanent link to our search query and input data is here: https://web.archive.org/web/20220701143537/https:/www.youtube.com/user/TEDxTalks/search?query=blockchain). We obtained 506 videos and selected 355 videos with titles and descriptions in English. For each selected video, we gathered initial statistics: publication date, number of views, “likes”, and comments. Fig 2 shows the distribution of views, likes, and comments.

The chosen visualization is called “*Violin plot*”, and it allows to visualize the distribution of a numeric variable for one or several groups. Violin plots are well adapted for large datasets, as stated on data-to-viz.com.

- Views range from 100 (10^2) to 2M (10^6,3), with a median value of some 15’000 (10^4,2)
- “Likes” range from 1 (10^0) to 300’000 (10^5,5), with a median value of 250 (10^2,4)
- Comments range from 1 (10^0) to 12’000 (10^4,1), with a median value of 13 (10^1,11)

We can see that there is one like for every 60 views (15’000/250) and one comment for every 19 “likes” (250/13).

### Data transformation

We also obtained some textual information about the content for each video: title, description, and automatic caption. The collection of the 355 titles, descriptions, and captions is called a *corpus*. Software for text analysis segments texts in a corpus into tokens (words or sentences) by word boundaries.

Table 1 shows the data of a different video in each row. Each column has a keyword that might appear in the video’s title, description, or caption. For example, we can see that the first and third videos discuss finance and music; there is no mention of companies in the last video. The frequency for that column is 0.

**Table 1:** Extracting keywords (“tokens”) from five YouTube videos about blockchain.

_id	financial	revolution	music	trust	company	world
Blockchain—The Engine of the Next Financial Revolution | Mauro Casellini | TEDxVaduz.1	8	2	1	20	5	4
Blockchain: Massively Simplified | Richie Etwaru | TEDxMorristown.1	1	0	2	61	6	9
What is blockchain and how can it change our society? | Ali Raza Dar | TEDxFHNW.1	1	0	0	2	1	6
Helping Refugees with Blockchain | Niall Dennehy | TEDxTrinityCollegeDublin.1	2	1	2	13	5	8
Blockchain, Art and the Metaverse | Cyrus James Khan | TEDxChiangMai.1	0	0	0	0	0	6

By analyzing the keywords used in the videos with the highest number of “likes” and comparing them with the keywords in the other videos, we perform a “*keyness analysis*.” We observed that the most liked videos use the keywords associated with the use of the technology (applications), such as “*trust*, *record*, *credit*, *house and ledger*”, more often than the others. Whereas the videos with the least number of “likes” tend to include more keywords associated with the technology itself and some fields of applications such as *“blockchain*, *bitcoin*, *transparency*, *health and transactions*.*”*

**Fig 3:**
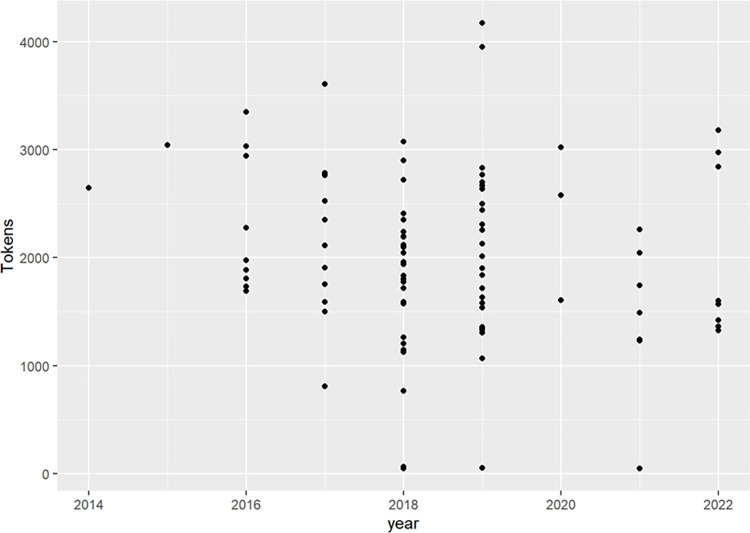
The 355 YouTube videos as points at the crossroad between publication year and the number of keywords (= tokens) used.

Then, analyzing the publication date and the number of keywords (also known as “tokens”) used in each talk makes it possible to visualize each presentation as a dot in Fig 3.

Accordingly, there is only one TEDx presentation in 2014 given by Charles Hoskinson (co-founder of the blockchain engineering company Input Output, the Cardano blockchain platform, and a co-founder of the Ethereum blockchain platform) that is associated with 2646 keywords. The only TEDx presentation given in 2015 by Rick Falkvinge (a Swedish entrepreneur and founder of the Swedish Pirate Party) is associated with 3039 keywords; it covers a larger spectrum of subjects. In 2018 some presentations focused on a few terms (where the total was ~4000 terms), whereas others tried to cover as much ground as possible. In 2020 there were few presentations, perhaps due to the pandemic, and they were more focused than the previous year. This could be a sign that the community is about to converge toward some central themes, as suggested by the notion of fashion waves of Baskerville and Myers [17].

### Data mining

To better understand how the keywords are used over time, we sorted them into topics. Topic modeling is a so-called unsupervised classification technique for machine learning. In other words, the software tries to sort the videos into subgroups called “topics” by combining the videos with keywords in common. To obtain our topics, we used a topic modeling technique called structural topic modeling [74,75]. Structural topic modeling can define topics that maximize the prevalence of a topic with respect to others. In our case, the prevalence was defined as the sum of the score of the video, the number of comments, and the year of publication. Table 2 shows the keywords associated with each topic. For every topic modeling algorithm, the choice of the number of topics is critical since it impacts the algorithm’s performance. After having explored different numbers of topics, we chose nine topics since that number strikes a nice balance between the need for semantic coherence (the most probable words in a given topic frequently co-occur together) and for the exclusivity of words to topics (few words should appear in more than one topic).

**Table 2:** Gathering keywords (“tokens”) into topics.

Topic 1	Topic 2	Topic 3	Topic 4	Topic 5	Topic 6	Topic 7	Topic 8	Topic 9
things	human	water	power	money	space	future	bitcoin	blockchain
looking	society	health	identity	currency	build	education	network	block
started	basic	energy	control	rules	machine	university	dollars	technologies
Remember	media	local	change	cryptocurrency	design	industry	computer	transactions
school	social	impact	organization	asset	problems	virtual	Price	smart

To better understand how topics are used, Table 3 shows how the system assigned topics to the first eight videos.

**Table 3:** Example of how the system assigns a topic (“tokens”) into topics.

Video	topic_keywords	gamma
1—Blockchain—The Engine of the Next Financial Revolution | Mauro Casellini | TEDxVaduz	9) blockchain, block, technologies, blockchain_technology, chain	0,47
	5) money, currency, rules, cryptocurrency, asset	0,32
2—Blockchain: Massively Simplified | Richie Etwaru | TEDxMorristown	9) blockchain, block, technologies, blockchain_technology, chain	0,33
	1) things, looking, going, started, little	0,33
3—The Energy Internet: How Blockchain Brings Power Back to the People | James Eggleston | TEDxFremantle	3) water, health, energy, local, impact	0,44
4—The Convergence of Blockchain, Machine Learning, and the Cloud | Steve Lund | TEDxBYU	6) space, build, machine, design, problems	0,39
5—How Blockchain Can Rebuild the Copyright Industry? | Charles Cheng | TEDxWoodside	7) future, education, university, industry, virtual	0,35
6—Blockchain-based Identity Could Save Democracy | Arwen Smit | TEDxErasmusUniversityRotterdam	4) power, identity, control, change, organization	0,49
7—How Can We Sustainably Power a Cryptocurrency Future? | Tara Shirvani | TEDxCambridgeUniversity	8) bitcoin, called, network, dollars, computer	0,33
	3) water, health, energy, local, impact	0,31
8—Fight fake news with blockchain: How image verification defends truth | Mounir Ibrahim | TEDxPenn	2) human, society, basic, media, social	0,36
**Videos**	**topic_keywords**	**gamma**
1—Blockchain—The Engine of the Next Financial Revolution | Mauro Casellini | TEDxVaduz	9) blockchain, block, technologies, blockchain_technology, chain	0,47
	5) money, currency, rules, cryptocurrency, asset	0,32
2—Blockchain: Massively Simplified | Richie Etwaru | TEDxMorristown	9) blockchain, block, technologies, blockchain_technology, chain	0,33
	1) things, looking, going, started, little	0,33
3—The Energy Internet: How Blockchain Brings Power Back to the People | James Eggleston | TEDxFremantle	3) water, health, energy, local, impact	0,44
4—The Convergence of Blockchain, Machine Learning, and the Cloud | Steve Lund | TEDxBYU	6) space, build, machine, design, problems	0,39
5—How Blockchain Can Rebuild the Copyright Industry? | Charles Cheng | TEDxWoodside	7) future, education, university, industry, virtual	0,35
6—Blockchain-based Identity Could Save Democracy | Arwen Smit | TEDxErasmusUniversityRotterdam	4) power, identity, control, change, organization	0,49
7—How Can We Sustainably Power a Cryptocurrency Future? | Tara Shirvani | TEDxCambridgeUniversity	8) bitcoin, called, network, dollars, computer	0,33
	3) water, health, energy, local, impact	0,31
8—Fight fake news with blockchain: How image verification defends truth | Mounir Ibrahim | TEDxPenn	2) human, society, basic, media, social	0,36

**Fig 4:**
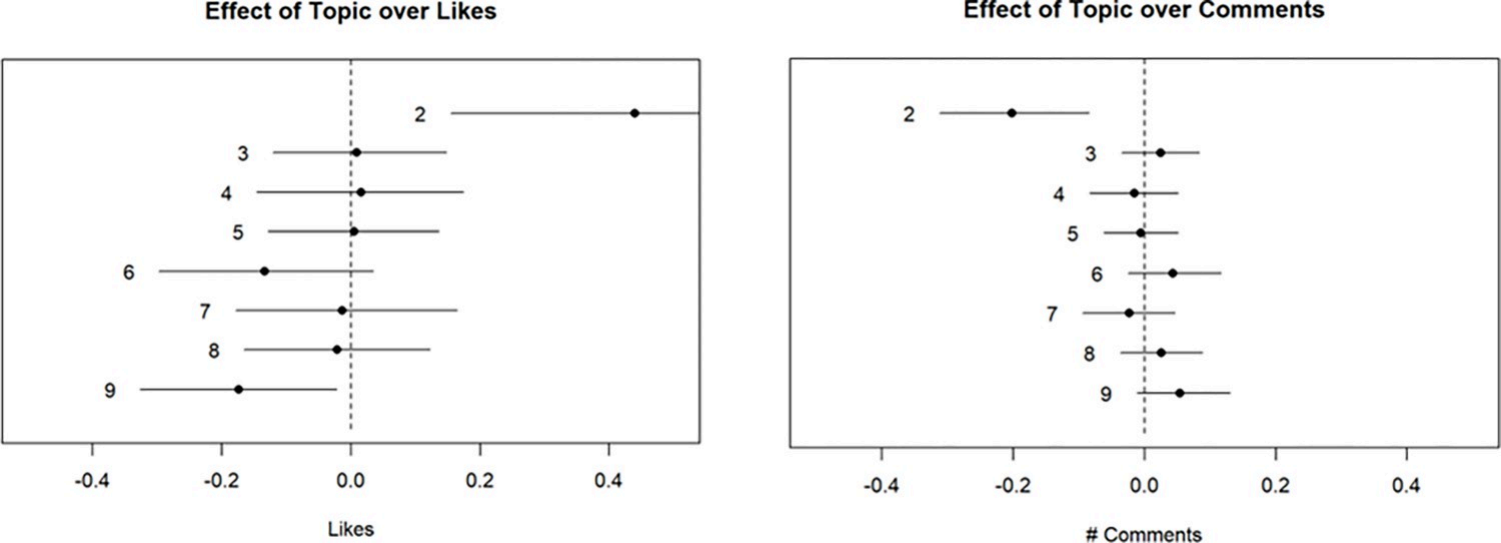
Effect of the chosen topic over the number of “likes” and comments of a TEDx Talk video about blockchain. For example, videos assigned to Topic 2 have more “likes” and fewer Comments than those assigned to Topic 9.

The degree of certitude that the system has is described by the gamma. Hence, the system believes that the first video falls in category 9, which focuses on the technology side of blockchain, even if the video appears to be about money and cryptocurrency. The second video deals with blockchain technology (topic 9) and uses simple words from topic 1 to explain the concept. If we observe the distribution of “likes” and comments that each topic has (Fig 4), we can identify which topics have the most significant amount of “likes” and comments. Accordingly, topic 2 seems to be associated with a greater number of “likes” but a smaller number of comments, whereas videos associated with topic 9 have, on average, fewer “likes” and spark a more intense conversation on YouTube with a greater number of comments.

**Fig 5:**
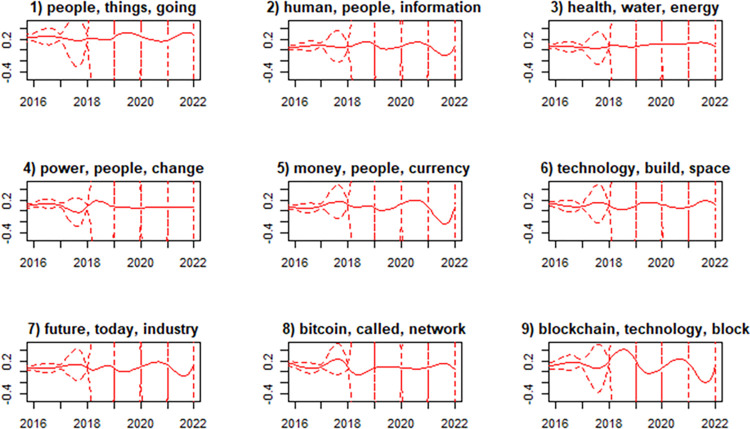
Evolution of each topic over time.

Finally, in Fig 5, we observed how videos on each topic are distributed over time. Most topics have a distribution of topics that is steady over time, except for topics 2 and 8, which had slightly more videos in 2016/2017 than in 2021/22, and topic 9, which had significantly fewer videos in 2016 but had multiple waves afterward.

## Discussion and conclusive remarks

In this paper, we have analyzed how blockchain has been discussed, presented, and eventually pitched by speakers invited to give a TED, or TEDx Talk, a relevant broadcaster of management ideas and technology innovation. The study presented in this paper contributes to understanding the relationship between the communication of technological innovation like blockchain and its adoption. This communication may capture not only the attention of practitioners or the public but also influence its eventual application and deployment. Moreover, In the case of blockchain, we argue that this understanding is relevant, considering that state-of-the-art research has shown that when looking at its application and deployment in domains other than cryptocurrency or the nascent non-fungible tokens (NFTs), blockchain technology has not been fully adopted or is still in its early stages, despite the hype the technology has received in the media and from technology advisors.

Furthermore, this paper provides a contribution also to the literature on management fashion, specifically focusing on technology innovation and communication through digital channels. In particular, the study shows how data-driven approaches provide structured insights on broadcasting and promoting management innovations, new practices, and technologies.

Looking at our results, for example, if we consider the degree of attention on a continuum from “*that’s interesting*” (represented in our case by the number of “likes”) to an engagement in commenting about the technology (represented in our case by the number of “comments”), we can see that the use of the technology seems to be associated with a more significant number of “likes” but with a smaller number of comments (as summarized by the keywords on Topic 2 of Table 2). In contrast, videos associated with the technology itself (as outlined by the keywords on Topic 9 of Table 2) have, on average, fewer “likes” but spark a more intense conversation amongst viewers. On the one hand, this could provide insights into the meaning of the “hype” related to blockchain, which is still not associated with an engagement in commenting on the use of the technology in the different application domains promoted in the TEDx Talks analyzed. On the other hand, this shows how technical issues related to blockchain have not captured the interest of a larger public of viewers because they are still related to a narrow understanding of the technology itself and not necessarily of what is needed for its application and use. This means that blockchain requires an advanced and narrow mastering of its technical features and not practical, high-level knowledge of them suitable to support blockchain’s use in a specific domain by an informed but still non-specialized user. This trend seems to follow the variants that have represented blockchain in the different fashion waves exhibited by the TED and TEDx Talks other the years, with a pattern of massive interest in any new blockchain technology fashion wave by the large public of viewers, but with an engagement in comments about their technicalities only for a minority of viewers. It is worth noting that the state-of-the-art literature on the bibliometrics of the blockchain shows a similar pattern. The main focus of the academic work is on the technical aspects, with some notable exceptions with the term “energy” and concepts from management like “business model.” Computer science and engineering emerge as the academic fields with the most contributors to blockchain technology. It appears then that more discussion about blockchain needs to be had before a common understanding of both its essential technical aspects and its potential applications is understood by the general public. TEDx events can be seen as a means of contributing to a greater understanding of blockchain over time. In a way, these talks take a grassroots approach to disseminating information about blockchain by leveraging local experts’ knowledge.

Besides the potential contribution of the research presented in this paper, the research aims to provide a contribution to practice. In particular, managers in charge of the adoption of new technology can find in our data-driven analysis a way to distinguish the different perspectives brought by broadcasters like TED, where a technology like the blockchain can be overshadowed in its evolution and application by the fact the focus is shifted on products and services built on it. Also, considering the insights of this study as an example, managers can evaluate the degree of attention and expertise they require for each fashion wave of new or emergent technology innovations.

Finally, we have identified four main limitations of our multimethod research, especially for data-driven analysis:

1. *The number of “likes” and comments as performance indicators* could be biased. Indeed, the publication date might influence the number of “likes”. Older videos have more views and more “likes” in absolute terms. It would be interesting to implement another performance indicator, such as the one used by the Internet Movies Database (IMDb). Nonetheless, using a composite measure as a dependent variable for prediction requires a level of analysis that goes beyond the scope of this paper.
2. *The number of topics can influence the results*. Indeed, it would be possible to use a more significant number of topics to have more granular results. Nonetheless, more topics would lower the semantic coherence of the keywords, making it harder to make sense of what each topic is about.
3. *An analysis of captions beyond the titles and descriptions* of the YouTube videos could provide additional insight. Most of the captions have been automatically generated by YouTube and are not 100% reliable. This can influence the overall result. Therefore, it might be worthwhile to analyze only the titles and descriptions of each video and compare the new results afterward with those presented here.
4. *A content analysis of the comments* would also shed light on what ideas are being honed and discussed by the viewing public. This analysis would allow for a clearer understanding of how ideas are circulating on the TEDx YouTube channel over time but also across talks.

Finally, the analysis conducted here focuses only on TEDx Talks found on the dedicated YouTube channel. To better grasp the amplitude of the management fashion of blockchain, future research should investigate other media sources. This would allow us to have a broader perspective on how blockchain is being talked about and whether there is any coherence in what is being talked about. In addition, given the high impact of official TED Talks, it would be worthwhile to compare these talks with other high-impact talks. This would also improve our understanding of the blockchain discourse circulating in the public sphere.
